# Stress Monitoring of Segment Structure during the Construction of the Small-Diameter Shield Tunnel

**DOI:** 10.3390/s23198023

**Published:** 2023-09-22

**Authors:** Liang Ding, Yi-Jie Sun, Wen-Zhi Zhang, Gang Bi, Hong-Zhong Xu

**Affiliations:** 1 North China Municipal Engineering Design & Research Institute Co., Ltd., Tianjin 300074, China; 2 College of Transportation Science and Engineering, Nanjing Tech University, Nanjing 210009, China; gbi@njtech.edu.cn (G.B.); hzhxu@njtech.edu.cn (H.-Z.X.); 3 Nanjing Municipal Engineering Management Office Construction Development Co., Ltd., Nanjing 210042, China

**Keywords:** small-diameter shield tunnel, stress, fiber Bragg grating, monitoring

## Abstract

Segmental stress during the construction process plays a pivotal role in assessing the safety and quality of shield tunnels. Fiber Bragg grating (FBG) sensing technology has been proposed for tunnel segment stress monitoring. A laboratory test was conducted to validate the reliable strain measurement of FBG sensors. The field in situ monitoring of a sewerage shield tunnel was carried out to examine the longitudinal and circumferential stresses experienced by the segments throughout the construction phase. The cyclic fluctuations in stress were found to be synchronized with the variations in shield thrust. A comparison was made between the longitudinal and circumferential stress variations observed during the shield-driving and segment-assembly processes. Additionally, the time required for the grouting to reach its full curing strength was estimated, revealing its impact on the stress levels and range of the pipe segment. The findings of this study offer an enhanced understanding of the stress state and health condition of small-diameter shield tunnels, which can help in optimizing the design and construction process of tunnel segments, as well.

## 1. Introduction

In recent years, China has actively pursued the construction of various infrastructure projects, such as subways, sponge cities, and underground integrated corridors, in response to rapid economic development and societal demands. In this context, shield tunneling has gained prominence, due to its efficient and expedited construction process, as well as its minimal disruption to surface traffic and adjacent structures. Consequently, there is a growing utilization of shield tunneling in the construction of sewage pipelines [1,2].

In contrast to conventional subway shield tunneling, sewerage tunnels typically have smaller diameters and experience relatively higher stress concentrations. The construction process of shield tunneling significantly impacts the segment, which serves as the primary load-bearing structure for tunnel support [3]. This impact is influenced by various construction factors, including the assembly load, grouting pressure behind the wall, and jack thrust, resulting in a complex mechanical state. Inadequate construction methods frequently lead to substantial alterations in the stress state of pipe tunnel segments [4]. Numerous empirical observations have demonstrated that tunnel segment damage frequently arises during the shield construction phase. Consequently, the assessment of mechanical performance variations in shield pipe segments during tunnel construction represents a pertinent challenge in the realm of pipe segment design and construction. In this regard, the implementation of tunnel in situ monitoring facilitates a comprehensive comprehension of stress alteration patterns in segments throughout the construction process [5].

Various techniques have been utilized to monitor the cross-section of tunnels, such as extensometers, angular encoder devices, total stations, photogrammetric methods, and ground three-dimensional laser scanning technology [6,7,8,9,10,11]. Additionally, two main methods are employed for the stress monitoring of shield tunnel segments: embedding reinforcement strain gauges within the segment during prefabrication, and installing strain gauges on the surface of the segment after construction. Traditional stress monitoring techniques, such as resistance, inductance, and vibrating wire sensors, are easily affected by environmental factors, which limits their effectiveness in long-term online monitoring for shield tunnels. In contrast, fiber-optic sensing (FOS) technology is a relatively novel monitoring approach developed in recent years that has several advantages, such as a compact size, immunity to electromagnetic interference, precise quantitative data, and the capability for continuous real-time automated tracking. The application of fiber-optic sensors in tunnel structural health monitoring has garnered significant attention in the research and has been the focus of dedicated research efforts [12]. To date, FOS technology has been successfully employed in various tunnel engineering projects, including the Bai Ni-jing No. 3 tunnel [13], Singapore subway tunnel [14], London Royal Post cast-iron tunnel [10], Barcelona TMB L-9 metro tunnel [15], and Suzhou Metro Line 1 subway tunnel [16,17].

Previously, there has been a lack of comprehensive research on the analysis of on-site monitoring data pertaining to the continuous stress experienced during the driving and assembling of small-diameter shield tunnels. This paper, however, employs FBG sensing technology to enable the real-time stress monitoring of sewerage tunnel segments in situ. The objective is to investigate the progression of segmental stresses throughout shield tunneling construction, thereby enhancing our understanding and comprehension of the structural response behavior. This knowledge can subsequently be utilized to evaluate the health condition of the tunnel and to optimize its design, ultimately leading to the creation of safer structures.

## 2. Principle of FBG

A fiber Bragg grating (FBG) is fabricated through the use of a phase mask to create near-field diffraction effects that generate spatial interference patterns in the optical fiber core. This modulates the refractive index periodically, forming the fiber grating structure. An FBG exhibits a good wavelength selectivity that satisfies the Bragg diffraction condition at a specific wavelength *λ*_B_. Light at *λ*_B_ that is incident on the FBG undergoes coupled reflection, while other wavelengths transmit through, unaffected. This selective reflection gives rise to a peak centered at *λ*_B_ on the reflection spectrum.

Changes in both the temperature and strain around the FBG sensor can cause a center wavelength shift Δ*λ*, and they satisfy the relationship equation

(1) $$\frac{\Delta \lambda }{\lambda_{B}}=C_{\varepsilon }\varepsilon +C_{T}\Delta T$$

where ε is the strain of the sensor, Δ*T* is the temperature change, and $C_{\varepsilon }$ and $C_{T}$ are calibration constants. More details about the fundamentals and operating principles of FBG systems can be found in the literature [18,19].

## 3. Sensing Performance Test

**Figure 1:**
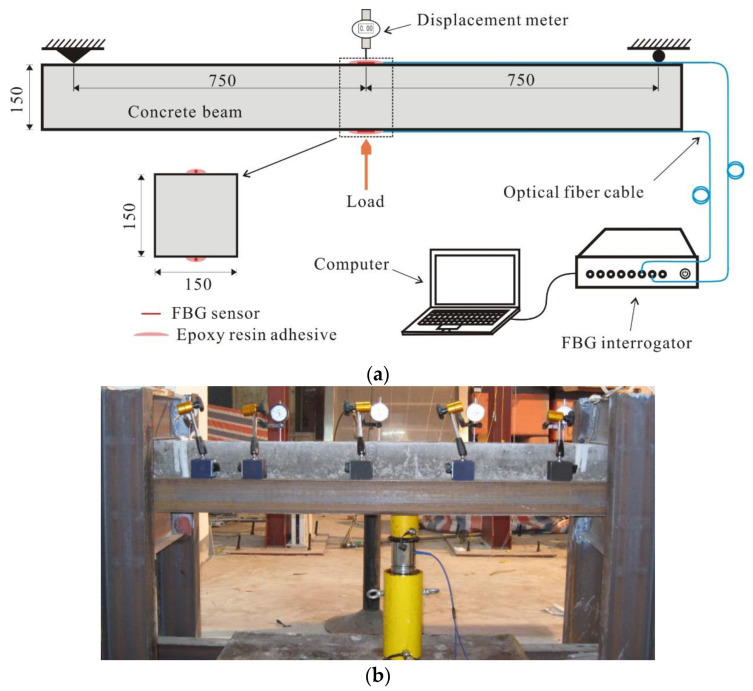
Schematic diagram of the loading experiment (unit: mm). (**a**) Diagrammatic sketch; (**b**) photograph.

In the FBG strain sensors, the fiber-optic sensing elements and the host material under test are separated by an intermediate packaging layer, resulting in the measured deformation being smaller than the actual value, due to the strain transfer loss [20,21,22]. In order to enhance the strain-sensing capabilities, survival ratio, and durability of FBG sensors during subsequent in situ testing, a high-strength epoxy resin adhesive was utilized for the direct attachment of uncoated FBG (bare fiber) onto the surface of a pre-fabricated shield tunnel segment [23]. To assess the impact of the strain transfer effectiveness and the potential measurement error, a three-point bending laboratory test was conducted on a concrete beam measuring 1.7 m in length and 15 cm × 15 cm as the cross-sectional area. The concrete beam used in the experiment possessed a strength grade of C50 and an elastic modulus of 3.45 × 104 MPa, which aligned with the shield tunnel segments present at the site. Two uncoated fiber Bragg grating (FBG) sensors were symmetrically affixed at the midpoint of the beam’s top and bottom surfaces, to monitor its strain response. The data were collected using a commercially available FBG interrogator (A04, Suzhou Nanzee Sensing Co., Ltd., Suzhou, China), which offered a strain measurement accuracy of ±2 με and an adjustable sampling frequency ranging from 1 to 100 Hz. Additional details regarding the laboratory test setup can be found in Figure 1.

**Figure 2:**
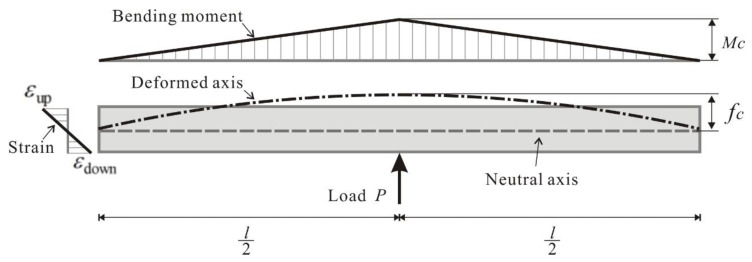
Schematic diagram of the concrete beam bending moment and deformation under loading.

Figure 2 depicts the force diagram of the beam structure when subjected to loading conditions. Where $M_{c}$ is the bending moment, $f_{c}$ is the deflection, and $\varepsilon_{\mathrm{up}}$ and $\varepsilon_{\mathrm{down}}$ are the axial strain on the upper and lower surfaces of the beam at the midpoint, while *P* represents the magnitude of the applied loading force. In accordance with the principles of mechanics of materials theory, these physical quantities adhere to the subsequent relationship [24,25].

(2) $$f_{c}=\frac{Pl^{3}}{48EI}$$

(3) $$M_{c}=\frac{Pl}{4}$$

(4) $$M_{c}=\frac{\varepsilon_{\mathrm{up}}-\varepsilon_{\mathrm{down}}}{b}EI$$

(5) $$\varepsilon_{\mathrm{up}}-\varepsilon_{\mathrm{down}}=\frac{12b}{l^{2}}f_{c}$$

where *E* is the beam modulus of elasticity. *I* is the cross-section moment of inertia. *b* is the beam cross-section height, and *l* is the beam length.

**Figure 3:**
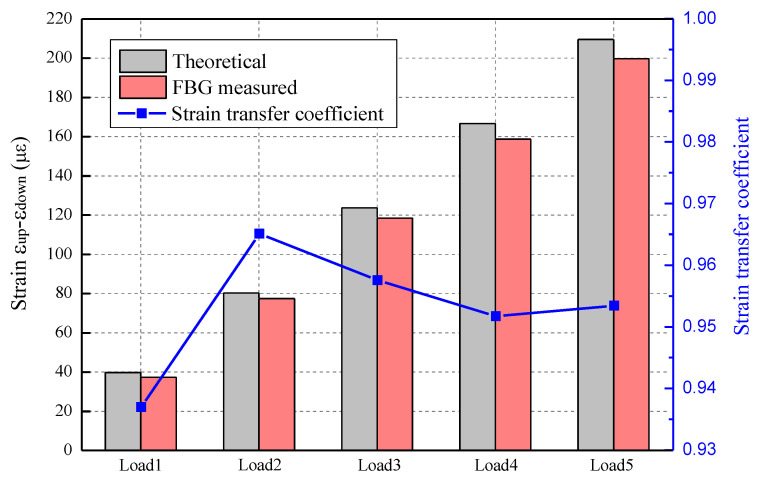
Comparison between the theoretical values and the strain measured via the FBG.

The theoretical beam bending strain can be determined via measuring the displacement at the midpoint of the beam during loading, as described in Equation (5). Alternatively, the actual values for the beam bending strain can be measured using FBG sensors positioned on the upper and lower surface of the beam. Figure 3 illustrates the disparity between the theoretical value and the strain data obtained from FBG measurements. It is observed that the strain value progressively increases with each load increment, while the ratio of FBG measured strain to theoretical strain (strain transfer coefficient) remains consistently stable across all load levels, with the average value being 0.955 and the minimum value being 0.94. These test results suggest that the estimated error in the actual strain measurement is effectively controlled within 6%.

## 4. Case Study

### 4.1. Site Description

**Figure 4:**
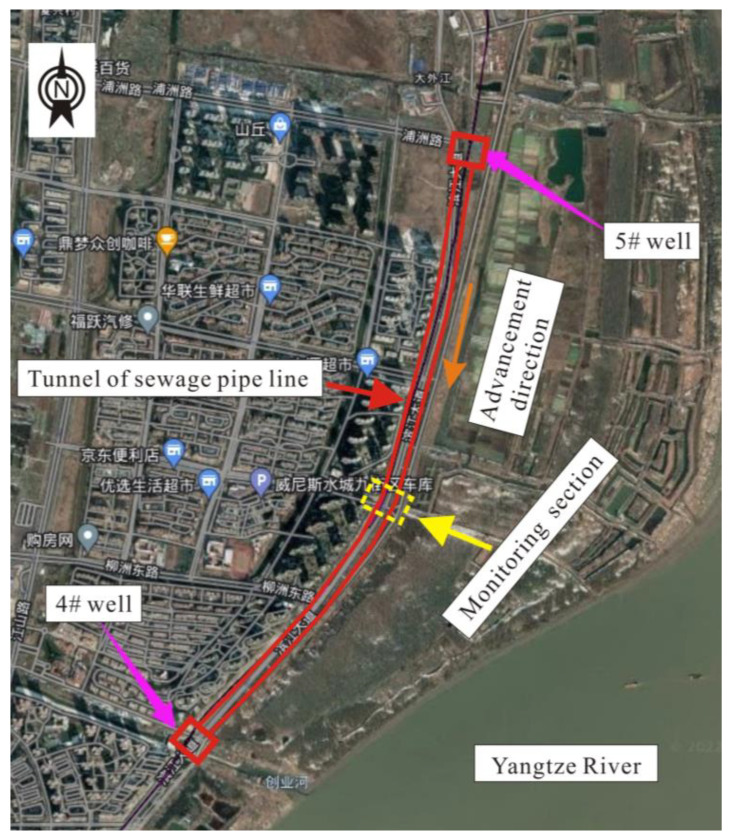
Plan of the site.

The site pertains to a specific segment of a sewerage tunnel project situated in Nanjing, Jiangsu Province, China. As depicted in Figure 4, the shield pipeline at this location commences at the 4# shield well and terminates at the 5# shield well, spanning a total length of 2433 m and buried at a depth of approximately 10–20 m. This project employs a small-diameter shield tunneling technique, featuring an inner diameter of 2.5 m and an outer diameter of 3 m. For the conduction of an in situ experimental investigation, a representative section of the shield tunnel was chosen, positioned in the middle of the pipeline and located 1348 m away from the 5# well.

The deformation and stress response of a tunnel segment were monitored during the progress of a shield. The field data offer valuable insights into the behavior of the tunnel cross-section during construction, which can serve as a reference for design and practice.

### 4.2. Installation of FBG Sensors

Each tunnel ring consists of five segments, namely A1, A2, B1, B2, and K. It was observed during the initial shield tunnel construction that significant deformation occurred when segment K (the splicing block) was positioned in the first and fourth quadrants.

**Figure 5:**
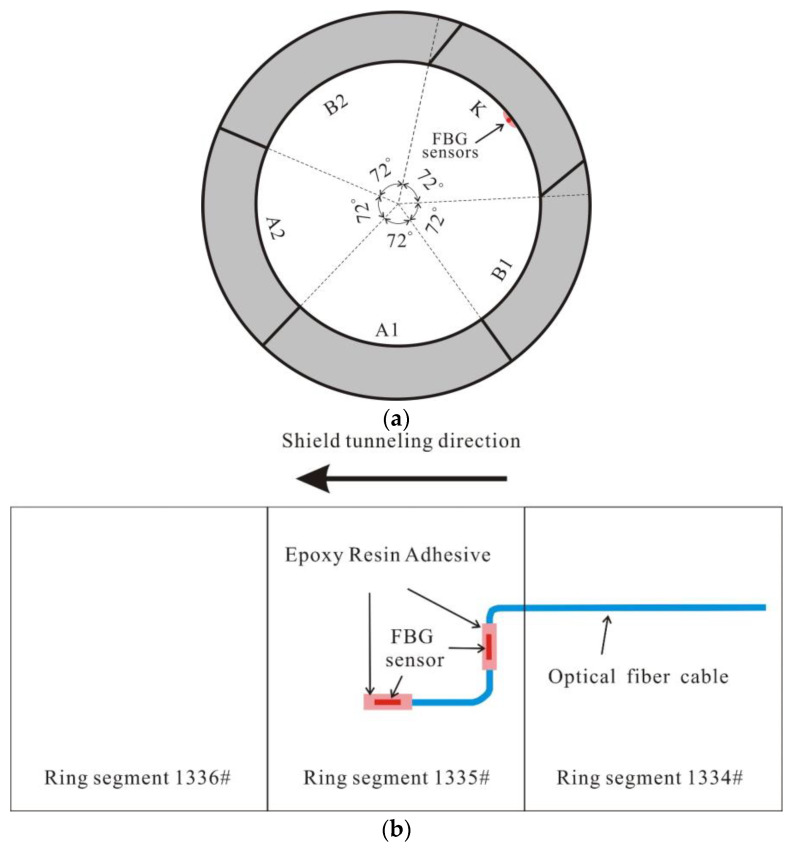
Installation details in the shield tunnel segment: (**a**) FBG sensors installed on the cross section; (**b**) FBG sensors installed along the longitudinal direction.

Hence, for the purpose of the field test, the monitoring point for the shield tunnel’s Ring 1335 was identified to be segment K. Taking into account the practicality of sensor placement during the actual construction process, the final arrangement of FBG sensors can be observed in Figure 5. To affix the sensors onto the concrete surface of the segment, a high-strength epoxy resin adhesive was utilized along both the circumferential and longitudinal directions.

**Figure 6:**
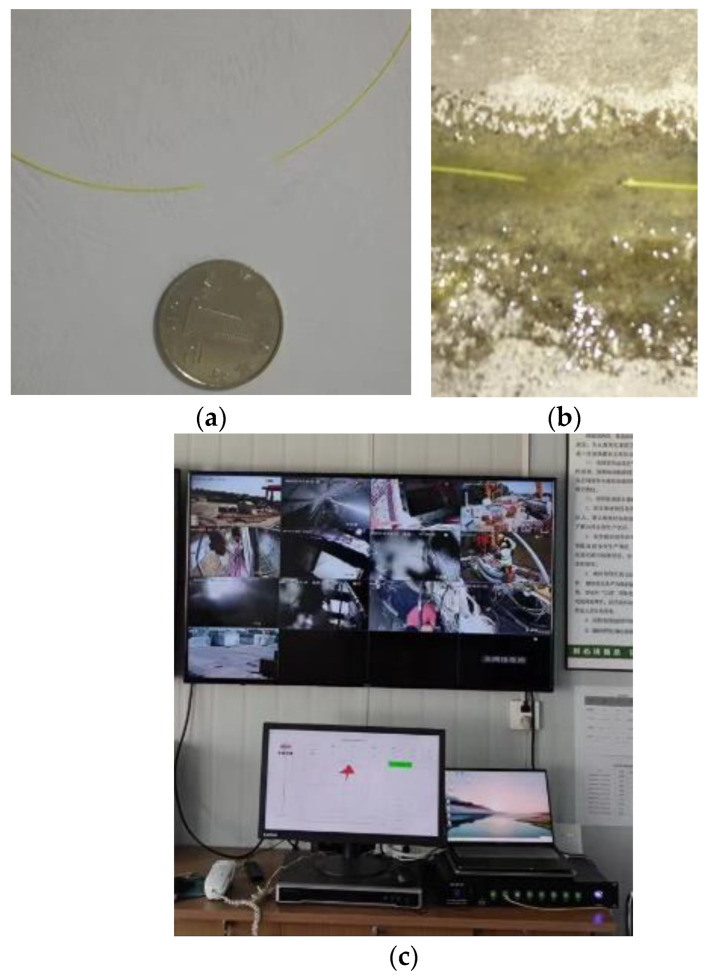
Photographs of the field installation and monitoring: (**a**) the FBG sensor; (**b**) the installation of the FBG sensor on the surface of the segment; (**c**) the monitoring console.

The FBG interrogator (A04, Suzhou Nanzee Sensing Co., Ltd., Suzhou, China) was positioned in the ground monitoring room and connected to the predetermined communication optical cable, thereby enabling remote online monitoring. Several in situ photographs are presented in Figure 6.

### 4.3. Measured Data and Analyses

#### 4.3.1. Construction Process of Shield Tunnel

**Figure 7:**
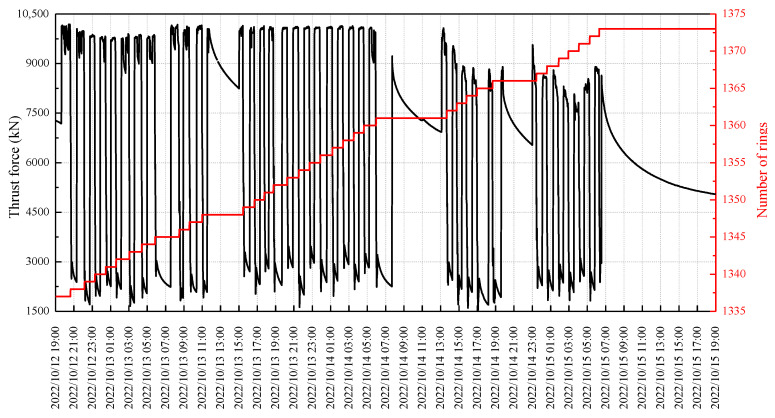
The thrust force and number of rings change over time.

Figure 7 illustrates the cumulative jacking forces exerted by the shield machine and the number of tunnel rings over time, with an average progressing rate of less than 2 h per ring. The sensors were installed between 18:00 and 18:50 on 12 October 2022. A monitoring period was conducted from 19:00 on 12 October to 19:00 on 15 October 2022, with a specific focus on the number of rings involved in shield tunneling, ranging from 1337 to 1373.

**Figure 8:**
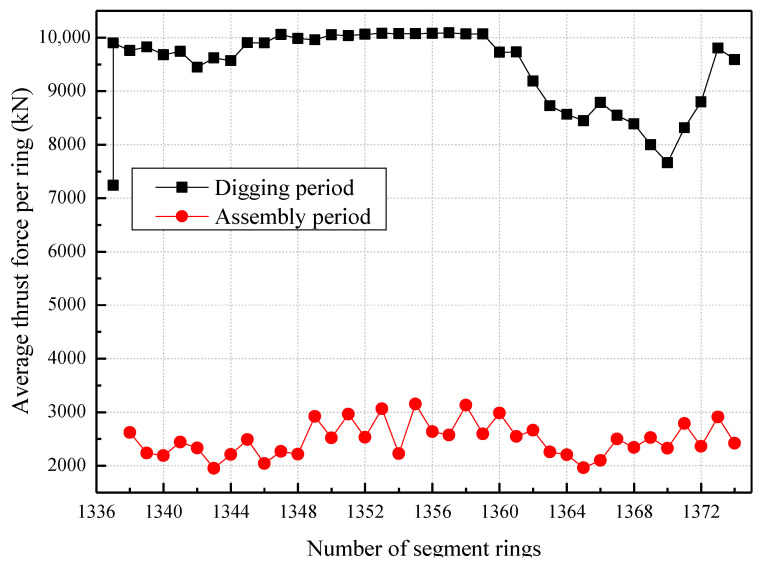
The tunnel thrust force varies with the number of rings.

By calculating the average thrust force values during the driving and assembling phases of each tunneling ring, the fluctuations in the shield thrust force can be observed with respect to the number of tunneling rings, as depicted in Figure 8. Throughout the shield driving process, the total thrust force consistently remained at a relatively elevated level, ranging from 7200 kN to 10,100 kN. Conversely, during the assembly of the segments, the thrust force exerted by the shield machine experienced a decline and fluctuation within the range of 1950–3200 kN.

#### 4.3.2. Monitoring Results of Tunnel Segment Strain

**Figure 9:**
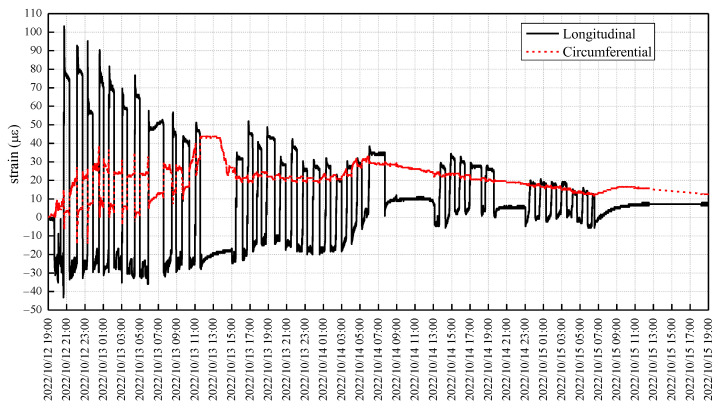
The strain monitoring results for the tunnel segment over time.

**Figure 10:**
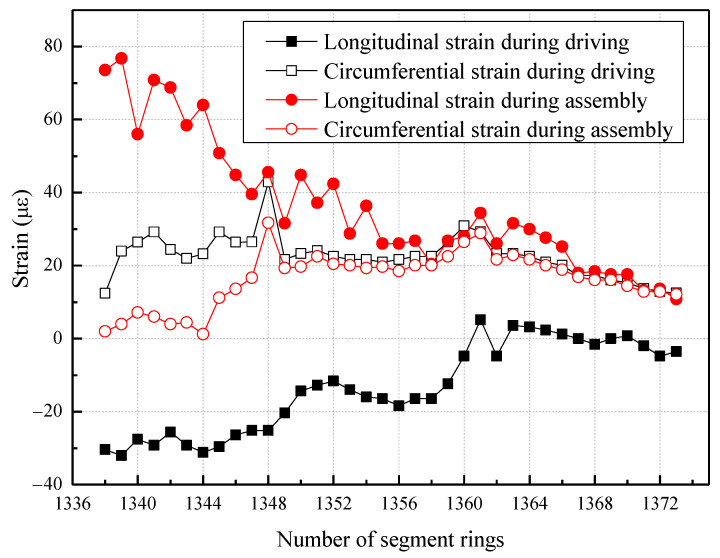
The strain of the tunnel segment varies with the number of rings.

The monitoring results for the circumferential and longitudinal strain of the tunnel segment are presented in Figure 9. Commencing at 19:00 on 12 October 2022, the sensing value at this point was designated as the initial baseline reading, serving as a reference for subsequent measurements. Through the subtraction of this baseline reading, the relative strain of the tunnel segment can be identified. In conjunction with Figure 7, the fluctuation in strain in the tunnel segment can be observed with respect to the number of tunneling rings, as illustrated in Figure 10.

Figure 10 demonstrates the periodic fluctuations in both the longitudinal and circumferential strain of the segments as the shield tunneling machine progresses. A comparison with Figure 8 reveals a direct correlation between the overall stress magnitude of the shield tunnel and the jack thrust. Throughout the driving cycle, the jacking forces intensify and remain in a state of high pressure. Consequently, the corresponding longitudinal compression of the segments increases (leading to an increase in negative strain), while the circumferential compression decreases (resulting in an increase in positive strain). During the assembly process of the shield tunnel segments, the jack thrust experiences a decrease and then remains in a state of low pressure. Consequently, the corresponding longitudinal compression of the segments decreases (resulting in an increase in positive strain), while the circumferential compression increases (leading to an increase in negative strain).

#### 4.3.3. Tunnel Segment Stress Analysis

The concrete utilized in the shield tunnel segments is of a strength grade of C50, with an elastic modulus $E_{c}$ of 3.45 × 10^4^ MPa and a Poisson’s ratio μ of 0.2. In accordance with the mechanical stress–strain relationship of materials, the circumferential and longitudinal stress of the segments can be mathematically expressed as follows.

(6) $$\sigma_{z}=\frac{E_{c}}{1-\mu^{2}}(\varepsilon_{z}+\mu \varepsilon_{\theta })$$

(7) $$\sigma_{\theta }=\frac{E_{c}}{1-\mu^{2}}(\varepsilon_{\theta }+\mu \varepsilon_{z})$$

where $\sigma_{z}$ is the axial stress, $\sigma_{\theta }$ is the hoop stress, $\varepsilon_{z}$ is the axial strain, and $\varepsilon_{\theta }$ is the hoop strain.

**Figure 11:**
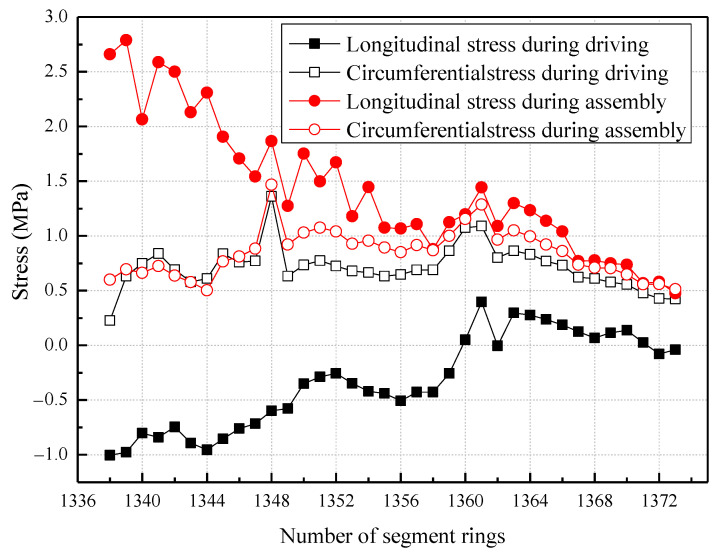
The stress of the tunnel segment varies with the number of rings.

The strain results for the segments depicted in Figure 10 are utilized to examine the variation in stress of the tunnel segments relative to their initial values as the number of rings increases, as demonstrated in Figure 11.

**Figure 12:**
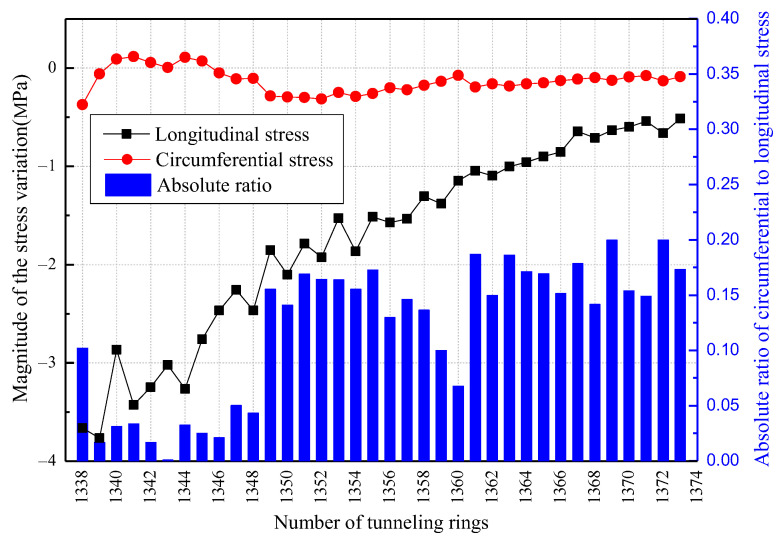
Comparison between the longitudinal stress and circumferential stress.

As stated in Section 4.3.2, the strain monitored in the tunnel segment is a relative variation in relation to the initial strain of the segment and, therefore, does not accurately depict the true stress conditions of the segment. To obtain a more accurate representation of the absolute stress response of the tunnel segment under changing constraints over time, it is recommended to calculate the difference in stress values between the segments during the driving cycle and assembling cycle of each ring. The magnitude of stress variation in the segments for each cycle can be determined as a function of the number of rings, as illustrated in Figure 12.

Figure 12 reveals that the cycling amplitudes of stress differ between the longitudinal and circumferential directions for the tunnel segmental, with the longitudinal stress being notably higher than the circumferential stress. The stress cycling state is related to the constraints around the segment, in addition to the thrust of the shield machine. Consequently, the shield tunnel segments primarily experience longitudinal stress during the construction process.

## 5. Discussion

**Figure 13:**
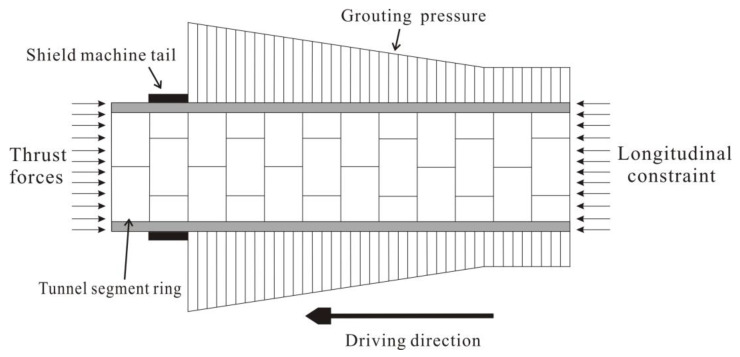
The load distribution along the longitudinal direction.

When the shield detaches, a circular gap is created between the soil and tunnel segments. The filling of this gap through synchronous grouting enables the segments to provide support to the ground. However, there is a time delay between the initial and final solidification of the grout, as it gradually gains strength. Additionally, the presence of groundwater and variations in the geological composition surrounding the tunnel can influence the process and timeline of grout curing, contingent upon the specific conditions. Prior to the complete hardening of the grout, the lateral constraint on the segments is relatively minimal. However, once the grout has fully cured, it envelops the segments entirely, resulting in a substantial reduction in the periodic fluctuation in the stress amplitude. The load distribution that the segment bears along the longitudinal direction is illustrated in Figure 13, which is derived from a theoretical analysis of the relevant literature [26,27], as well as the measurement findings presented in Section 4.3.

In relation to the longitudinal stress depicted in Figure 12, it can be observed that the relative amplitude of stress variation at the measurement points along the tunnel segments exhibits a general downward trend. During the driving cycle, the longitudinal compressive stress on the segments diminishes progressively as they move away from the shield machine, owing to the counteracting longitudinal frictional resistance between the segments and grout body in contrast to the thrust exerted by the shield jack. Conversely, during the assembling cycle, the axial constraint intensifies as the number of rings increases, leading to a decrease in the rebound of tunnel segments. Namely, the monitored fluctuation in the amplitude of the axial compressive stress also gradually diminishes.

In relation to the circumferential stress depicted in Figure 12, there is a slight disparity in the relative amplitude of stress variation trends. During the initial phases prior to complete curing, the grout exhibits a fluid-plastic state. The grouting pressure diminishes as the distance from the shield machine increases, leading to a convergence toward a steady value. Over time, the grout gradually solidifies, resulting in an enhanced and, ultimately, stable circumferential constraint. This aligns with the observed pattern of circumferential stress, which initially increases and subsequently decreases with the number of segments. Additionally, the cyclic stress ratio experiences a substantial alteration subsequent to ring 1348, signifying the attainment of maximum strength by the grout, which aligns with a curing period of approximately 24 h.

## 6. Conclusions

This research paper presents the implementation of FBG sensing technology for the in situ stress monitoring of small-diameter shield tunnel segments. The study investigates the stress and deformation characteristics during the construction process, leading to the following conclusions:

(1) The strain-sensing capabilities of FBG sensors have been verified through a three-point bending test conducted on a concrete beam. A comparison between the theoretical values and the strain data measured by FBG sensors reveals that the relative errors in the actual strain measurements are estimated to be within 6%.
(2) The longitudinal and circumferential relative stresses experienced by the tunnel segments undergo periodic fluctuations during the driving and assembling cycles. Notably, the cycling amplitudes of longitudinal stress are considerably higher than those of the circumferential stress. During the construction process, the small-diameter shield tunnel segments primarily experience longitudinal stress.
(3) The test results reveal that the periodic stress pattern indicates that the grout attains its maximum strength after approximately 24 h. Furthermore, the ratio of variation amplitude between the circumferential stress and longitudinal stress significantly diminishes once the grout has fully cured. It is worth noting that the grouting pressure applied to the tunnel segments has a substantial impact on the circumferential stress, with an influence range spanning six rings in the conducted test.

The section of the monitoring tunnel chosen for this project is situated deep underground and far away from the exit. Throughout the short-term monitoring period, the overall temperature variation in the environment remains relatively minimal, and its influence on the test outcomes has been disregarded. However, as time progresses, the stress exerted on the pipe segment due to construction gradually diminishes, while the temperature impact continues to intensify. Subsequent investigations will incorporate temperature monitoring strategies to enhance the precision and dependability of the monitoring findings.

## Data Availability

Not applicable.
